# Performance of Different Fibers in the Extraction Parameters of Volatile Compounds Present in Alecrim-Do-Campo (*Baccharis dracunculifolia*)

**DOI:** 10.3390/metabo16030149

**Published:** 2026-02-24

**Authors:** Lucas Silveira Garcia, Talvane Coelho, Afonso Henrique de Oliveira Júnior, Ana Luiza Santos Vieira, Mauro Ramalho Silva, Eduardo José Azevedo Corrêa, Ana Cardoso Clemente Filha Ferreira de Paula, André Mundombe Sinela, Delfina Fernandes Hlashwayo, Eric Marsalha Garcia, Hosane Aparecida Taroco, Júlio Onesio-Ferreira Melo

**Affiliations:** 1 Department of Exact and Biological Sciences, Universidade Federal de São João del-Rei, Sete Lagoas 35702-031, MG, Brazil; lucassg.bh@gmail.com (L.S.G.); coelhotalvane@gmail.com (T.C.); afonsohoj@gmail.com (A.H.d.O.J.); anavieiranutricionista@gmail.com (A.L.S.V.); eric@ufsj.edu.br (E.M.G.); hataroco@ufsj.edu.br (H.A.T.); 2 Departament of Nutrition, Pontifical Catholic, Pontifícia Universidade Católica de Minas Gerais, Belo Horizonte 30535-000, MG, Brazil; mauroramalhosilva@gmail.com; 3 Instituto Tecnológico de Agropecuária de Pitangui—ITAP, Empresa de Pesquisa Agropecuária de Minas Gerais, BR-352, km 35—Zona Rural, Pitangui 35650-000, MG, Brazil; edujcorrea@gmail.com; 4 Departamento de Ciências Agrárias, Instituto Federal de Educação, Ciência e Tecnologia de Minas Gerais, Campus Bambuí, Bambuí 38900-00, MG, Brazil; ana.paula@ifmg.edu.br; 5 Instituto Politécnico da Universidade José Eduardo dos Santos, Huambo 2458, Angola; andresinela@gmail.com; 6 Departamento de Ciências Alimentares, Universidade Eduardo Mondlane, Maputo CP 257, Mozambique; delfina.hlashwayo@uem.ac.mz; 7 Higher Institute of Agronomy, University of Lisbon, 1349-017 Lisbon, Portugal

**Keywords:** *Baccharis dracunculifolia*, chemical profile, volatile compounds, HS-SPME, Brazilian Savanna

## Abstract

**Background:** Alecrim-do-campo (*Baccharis dracunculifolia*) is a species of agroindustrial and medicinal relevance that has attracted increasing interest in recent years due to its distinctive chemical profile rich in bioactive compounds. In this context, the present study evaluated the efficiency of different extraction conditions for volatile compounds in alecrim-do-campo, aiming to contribute to the traceability of products that use this species as a source of metabolites. **Methods:** A 2^3^ factorial design was employed to assess the best conditions for extracting volatiles by headspace solid-phase microextraction (HS-SPME), using three different semipolar fibers (PDMS/DVB, DVB/CAR/PDMS and CAR/PDMS). Regarding the effect of the variation factors to which the samples were subjected, only the extraction time (min) had a significant effect on compound extraction using the CAR/PDMS fiber. **Results:** In total, 79 volatile compounds were detected using the three fibers, with CAR/PDMS (43 compounds) and DVB/CAR/PDMS (44 compounds) showing the highest diversity. The nature of this study is important for the industry because it optimizes the search for quality parameters in plant-derived products.

## 1. Introduction

Alecrim-do-campo (*Baccharis dracunculifolia*) is a species belonging to the Asteraceae family, widely distributed in South America, with marked agroindustrial importance due to its close relationship with propolis production, and, in traditional medicine, due to its pharmacological potential [1,2,3]. Compounds synthesized by alecrim-do-campo are strongly associated with antimicrobial activity against a range of medically important microorganisms, such as bacteria and fungi [4,5], as well as antitumor activity [6], cytoprotective, immunomodulatory [7], hepatoprotective [8] and photoprotective effects [9].

In addition to its economic relevance and experimentally validated bioactive properties, *B. dracunculifolia* has a long-standing history of use in folk medicine across several Latin American countries, particularly Brazil, Argentina, Paraguay, and Uruguay [10,11]. Ethnobotanical surveys and regional pharmacopeias report the use of leaves and aerial parts in infusions, decoctions, and topical preparations for the treatment of inflammatory disorders, gastrointestinal disturbances, hepatic disorders, wound healing, and infectious conditions [12].

Within the components of its complex chemical profile, several phenolic compounds, such as artepillin C and *p*-coumaric acid [11,12], and terpenoids such as α-pinene, trans-nerolidol, δ-elemene and α-muurolol have been reported [13,14,15,16]. In general, terpenoids play a crucial role in plant ecophysiology, contributing both to organism survival through indirect defense against herbivores and to reproduction, by attracting pollinators [17]. In addition to their ecological functions, in agroindustry terpenoids stand out not only for their bioactive properties but also for their organoleptic properties, such as distinct odor, which are important for product quality and consumer acceptance.

Conventional extraction techniques commonly applied to plant volatiles, such as hydrodistillation, steam distillation, and solvent-based extraction, are widely used but present important limitations, including long extraction times, high solvent consumption, thermal degradation of labile compounds, and possible loss of highly volatile constituents. In contrast, headspace-based techniques focus on the volatile fraction released from the sample matrix, minimizing matrix interference and reducing chemical alterations during extraction. These methodological differences have driven the increasing adoption of solvent-free, low-temperature approaches for the characterization of plant volatilomes, particularly when the objective is to preserve native chemical profiles and improve analytical sensitivity. As such, in the analysis of volatile compounds in plant species, headspace solid-phase microextraction (HS-SPME) is widely employed in the study of plant material. This method uses fibers coated with specific materials to adsorb analytes in the sample, and the choice of fiber coating and the definition of extraction parameters are decisive in the analytical process [18].

Coupled to this method, gas chromatography–mass spectrometry (GC-MS) is used as the analytical technique, enabling the separation of complex mixtures, compound identification and quantification. Separation of mixture components is based on the time each compound takes to pass through the chromatographic column. Subsequently, identification is achieved by ionizing and fragmenting these compounds in the mass spectrometer. Ionization and fragmentation allow analysis based on the mass-to-charge ratio of fragments, enabling compound identification [19].

Therefore, this study aimed to evaluate the efficiency of different extraction conditions for volatile compounds in alecrim-do-campo to support the search for biochemical markers of alecrim-do-campo.

## 2. Materials and Methods

### 2.1. Sampling and Sample Preparation

Samples were collected from a population in Sete Lagoas, Minas Gerais, Brazil, at an altitude of 761 m, at 19°27’57” S and 44°14’48” W. After collection, the plant material was placed in polyethylene bags and transported to the Phytochemistry Laboratory of the Federal University of São João del-Rei, Sete Lagoas Campus (UFSJ/CSL), where it was washed with running water to remove impurities.

### 2.2. Headspace Solid-Phase Microextraction Fibers

The extraction of volatile compounds was evaluated using three semipolar HS-SPME fibers: polydimethylsiloxane/divinylbenzene (PDMS/DVB, 65 μm), divinylbenzene/carboxen/polydimethylsiloxane (DVB/CAR/PDMS, 50/30 μm) and carboxen/polydimethylsiloxane (CAR/PDMS, 75 μm).

### 2.3. GC-MS Analysis Conditions

Analyses were performed on a gas chromatograph (GC-2010 Plus, Shimadzu) coupled to a GC/MS-QP2010 (Kyoto, Japan) SE mass spectrometric detector with an ion-trap analyzer and a split/splitless injector operated in splitless mode, installed at the Food Analysis Laboratory—UFSJ/CSL.

Helium was used as the carrier gas at a constant flow of 1 mL min^−1^. The chromatographic analysis conditions were as follows: injector temperature 250 °C; desorption time 5 min; ion source temperature 200 °C; interface temperature 275 °C. A capillary column RTx^®^-5MS (Crossbond® (Restek, Bellefonte, PA, USA) 5% diphenyl/95% dimethylpolysiloxane) was used with the following dimensions: 30 m length, 0.25 mm internal diameter and 0.25 μm film thickness (Restek, Bellefonte, PA, USA). Column oven temperature was programmed as follows: initial temperature 40 °C (3 min), then 7 °C min^−1^ to 125 °C (1 min isothermal), followed by 5 °C min^−1^ to 210 °C (1 min isothermal), and finally 15 °C min^−1^ to 245 °C, held isothermally for 3 min. Ionization of volatile compounds was carried out by electron impact (EI) at an energy of 70 electron volts (eV). Ion source and transfer line were maintained at constant temperatures of 250 °C and 280 °C, respectively, with the system operating under vacuum sustained by a turbomolecular pump. Scan rate was 3 Hz.

### 2.4. Factorial Design for Optimization of Volatile Organic Compounds (VOCs) Extraction

A 2^3^ factorial design with three replicates at the central point was employed with the aim of determining the optimal conditions to reach the partition equilibrium of analytes between the sample and the fiber in the headspace mode, yielding the highest recovery of volatile organic compounds after chromatographic analysis. The following independent variables were evaluated: extraction time (min), extraction temperature (°C) and sample mass (g), for the extraction of volatile organic compounds (VOCs) from alecrim-do-campo (Table 1). The factorial design was built using Matlab, version 7.9.0.529. The number of VOCs captured per run was used as the response variable for the optimization of the evaluated factors, at a significance level of *p* = 0.05.

As a standard procedure for conditioning the SPME fibers (PDMS/DVB, DVB/CAR/PDMS and CAR/PDMS), the fibers were conditioned in the gas chromatograph, according to the manufacturer’s recommendations for each fiber type. HS-SPME was used for the extraction of volatile organic compounds. Samples (0.5, 1.0 and 1.5 g) were weighed into 20 mL headspace vials, sealed with screw-cap aluminum lids and rubber septa. The headspace vials were preheated for 5 min at the temperature designated for each treatment (40, 50 or 60 °C) in a stainless-steel bath on a heating plate. Then, the SPME fiber was introduced in the headspace mode for analyte adsorption and exposed to the gaseous phase for the extraction time defined for each treatment. After extraction, the fiber was inserted into the GC injector at 250 °C for 5 min for desorption of the extracted volatile organic compounds [20,21].

### 2.5. Identification of VOCs

Peaks selected for identification were those with a slope of 1000 min^−1^ and a relative area greater than 0.05%. Compound identification was performed by comparing the obtained spectra with the NIST (National Institute of Standards and Technology) library, considering a similarity index (SI) higher than 85.

Volatile organic compounds (VOCs) were identified based on the mass-to-charge (*m*/*z*) ratio corresponding to each peak in the total ion chromatogram of each analyzed sample, which were compared with mass spectra obtained by electron impact (EI) ionization at 70 eV, with a full-scan range from 35 to 350 *m*/*z* [22,23,24,25]. To confirm VOCs, the identified compounds were compared with those previously reported in the literature. Signal-to-noise (S/N) ratios and peak intensities were obtained from the GCMSolution version 2.72 (Shimadzu, Kyoto, Japan) and exported to Microsoft Office Excel 2024, which was used for peak selection; in this work, only peaks with a relative area higher than 0.05% were retained.

## 3. Results

### 3.1. Optimization of Extraction Conditions for HS-SPME Fibers

The results from the 2^3^ factorial design with three central-point replicates are presented in Table 2, with the number of VOCs as the response variable. The number of extracted compounds varied from 41 to 75 for the CAR/PDMS fiber, from 21 to 67 for the PDMS/DVB fiber and from 39 to 74 for the DVB/CAR/PDMS fiber, considering the treatments with the lowest and highest numbers of compounds obtained, respectively.

**Figure 1:**
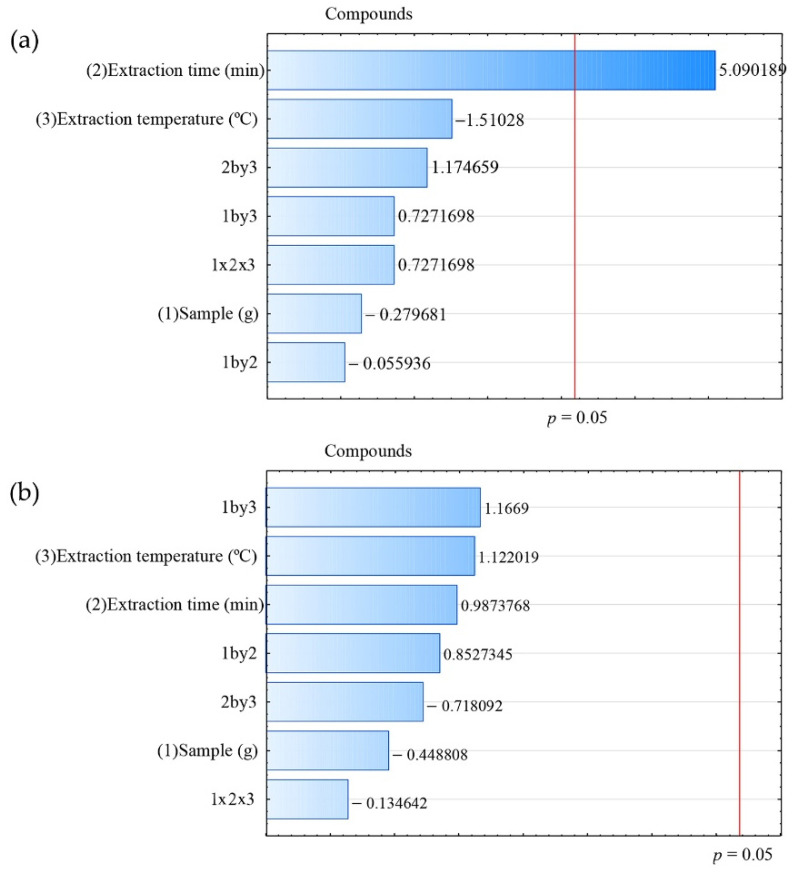

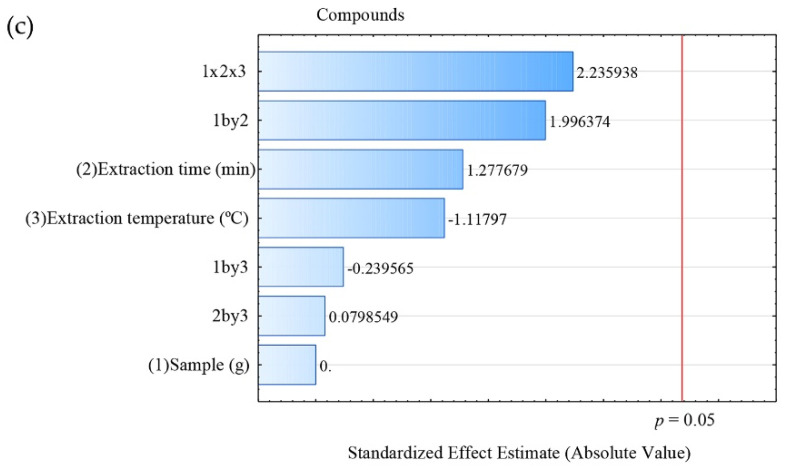
Pareto chart for the extraction factors of volatile compounds present in alecrim-do-campo, obtained using the fibers: (**a**) CAR/PDMS; (**b**) PDMS/DVB; (**c**) DVB/CAR/PDMS.

Pareto charts for each fiber (Figure 1) show that the factors that cross the red line corresponding to a significance level of *p* = 0.05 are considered statistically significant. Only fiber exposure time was statistically significant in the extraction process when using the CAR/PDMS fiber (Figure 1a).

Figure 1b shows the results for the PDMS/DVB fiber. For this fiber, no factor showed a statistically significant influence on compound extraction.

Figure 1c was generated from the data obtained using the DVB/CAR/PDMS fiber. Similarly to the PDMS/DVB fiber, no factor had a statistically significant impact on compound extraction.

**Figure 2:**
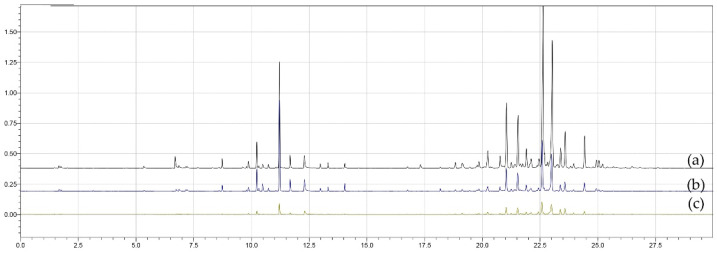
Superimposed chromatograms of treatments (**a**) T4, (**b**) T6 and (**c**) T9 obtained using the CAR/PDMS fiber in the extraction process.

Response surface plots illustrating the interaction among factors and the number of extracted compounds using the CAR/PDMS fiber are presented in Figure 2. Among the treatments, T4, corresponding to 0.5 g of sample, heating at 60 °C and extraction time of 15 min, yielded the highest number of compounds (75). In contrast, treatment T9, with 1.5 g of sample, heating at 60 °C and extraction time of 5 min, showed the lowest number of extracted compounds (Table 2).

In Figure 2a, it can be observed that the sample mass used did not markedly interfere with the number of extracted compounds. In Figure 2b, it can be inferred that, at temperatures close to 40 °C, smaller sample masses resulted in a lower number of identifiable compounds. In Figure 2c, it is evident that significant numbers of compounds can be obtained with extraction times shorter than 10 min, regardless of temperature.

Figure 2 presents superimposed chromatograms for treatments (a) T4, (b) T6 and (c) T9, obtained using the CAR/PDMS fiber. The chromatographic profiles differ in intensity and number of peaks, indicating variations in volatile composition among treatments, with T4 showing the highest number of compounds, T6 corresponding to the central point of the design, and T9 yielding the lowest number of extracted compounds.

**Figure 3:**
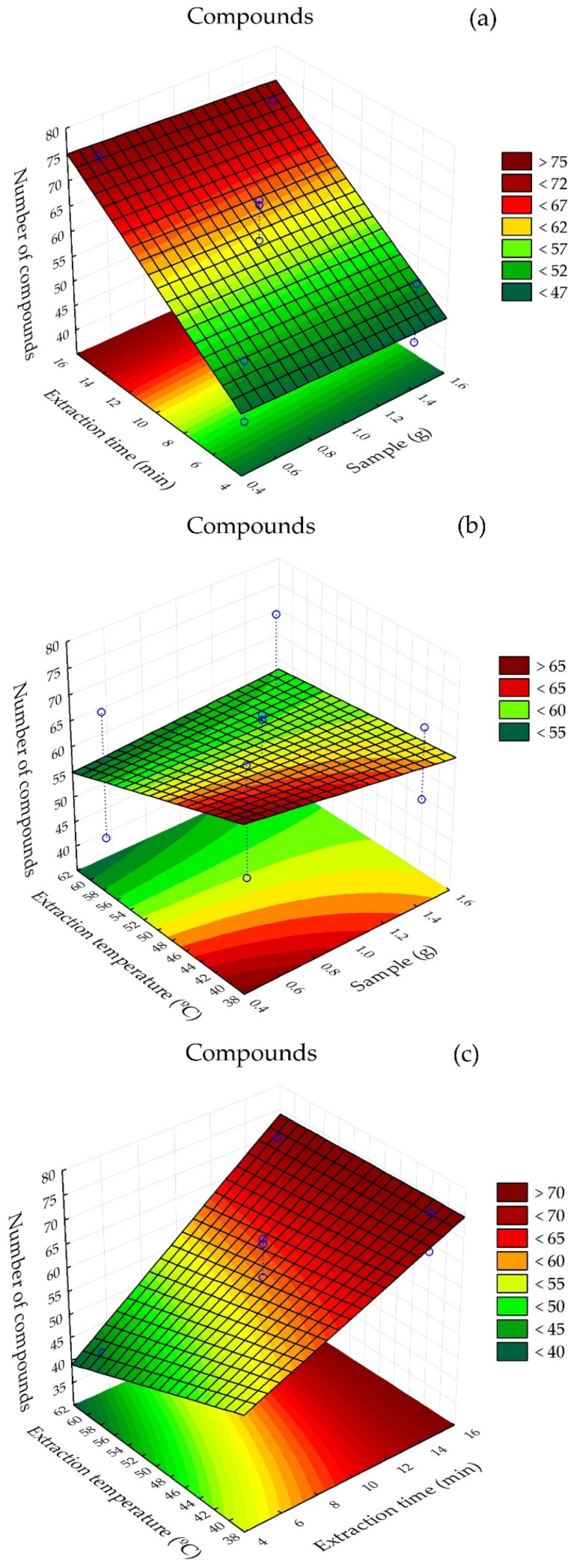
Response surface plot for volatile compounds present in alecrim-do-campo obtained using the CAR/PDMS fiber. (**a**) interaction between sample mass (g) and extraction time (min); (**b**) interaction between sample mass (g) and analyte heating temperature (°C); (**c**) interaction between fiber exposure time (min) and analyte heating temperature (°C).

For the PDMS/DVB fiber, the treatment that most stood out was T11, corresponding to 1.5 g of sample, heating at 60 °C and 15 min of fiber exposure, with 67 extracted compounds. The treatment that resulted in the lowest number of isolated compounds was T8, corresponding to 1.5 g of sample, heating at 40 °C and 5 min of extraction, yielding 21 compounds (Table 2). Response surface plots illustrating the interaction between factors and the number of extracted compounds are shown in Figure 3.

In Figure 3a, it can be seen that, for extraction times above 12 min, higher sample masses favored an increase in the number of extracted VOCs. In Figure 3b, it can be inferred that temperatures above 50 °C and sample masses greater than 1.0 g tended to yield more VOCs. Figure 3c shows that more compounds were extracted at temperatures above 55 °C and with extraction times of at least 12 min.

**Figure 4:**
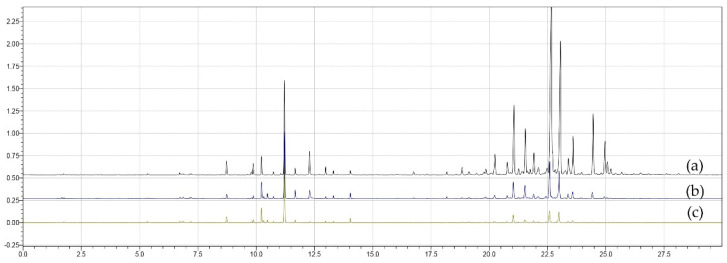
Superimposed chromatograms of treatments (**a**) T11, (**b**) T6 and (**c**) T8 obtained using the PDMS/DVB fiber in the extraction process.

Figure 4 presents superimposed chromatograms for treatments (a) T11, (b) T6 and (c) T8, obtained with the PDMS/DVB fiber. The chromatographic profiles showed variations in intensity and number of peaks, reflecting differences in volatile composition among treatments. Treatment T11 showed the highest number of extracted compounds, T6 corresponded to the central point of the experimental design, and T8 showed the lowest diversity of detected compounds.

**Figure 5:**
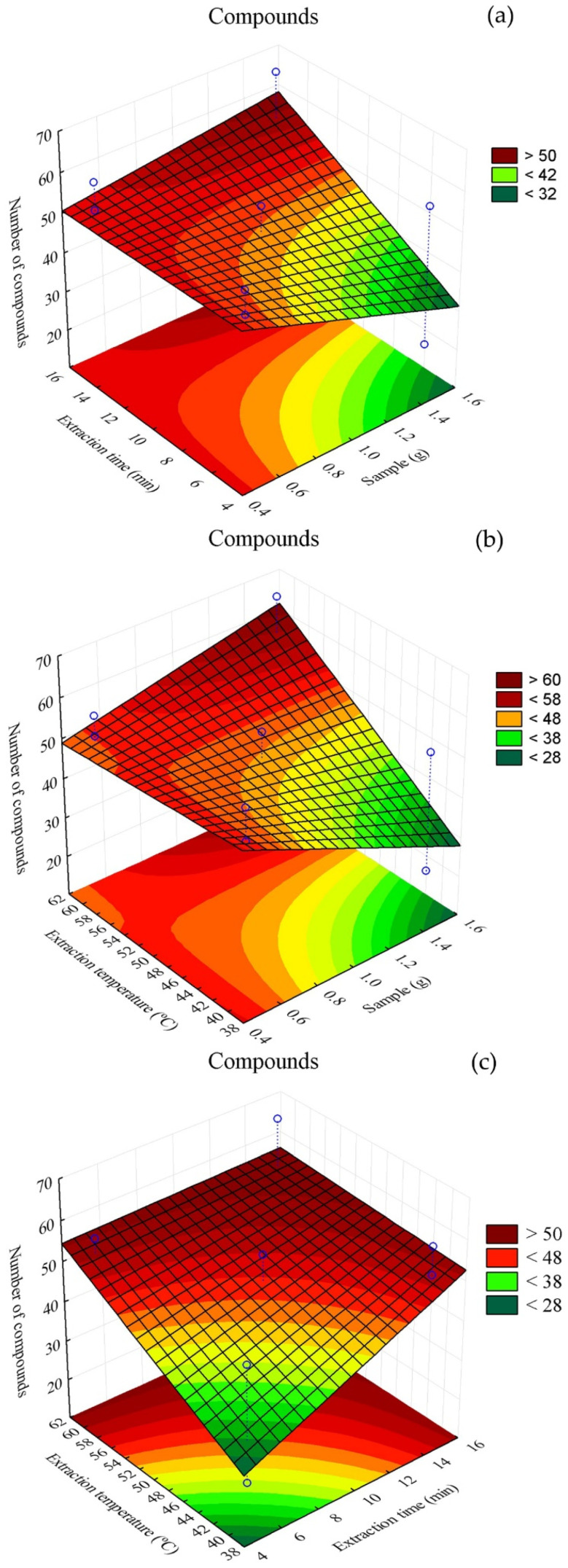
Response surface plots for volatile compounds present in alecrim-do-campo obtained with the PDMS/DVB fiber. (**a**) interaction between sample mass (g) and extraction time (min); (**b**) interaction between sample mass (g) and analyte heating temperature (°C); (**c**) interaction between fiber exposure time (min) and analyte heating temperature (°C).

For the DVB/CAR/PDMS fiber, the treatment that most stood out was T11, corresponding to 1.5 g of sample, heating at 60 °C and 15 min of extraction, with 74 extracted compounds. The treatment that resulted in the lowest number of extracted compounds was T9, corresponding to 1.5 g of sample, heating at 60 °C and 5 min of extraction, yielding 39 compounds (Table 2). Figure 5 shows the response surface plots illustrating the interaction among factors and the number of extracted compounds.

In Figure 5a, it can be seen that higher extraction was obtained with extraction times above 12 min and sample masses close to 1.5 g. In Figure 5b, it is observed that, at temperatures below 45 °C, regardless of sample mass, there was a tendency to extract more VOCs. Figure 5c shows that temperatures below 45 °C combined with 15 min of extraction favored an increase in the number of extracted compounds.

**Figure 6:**
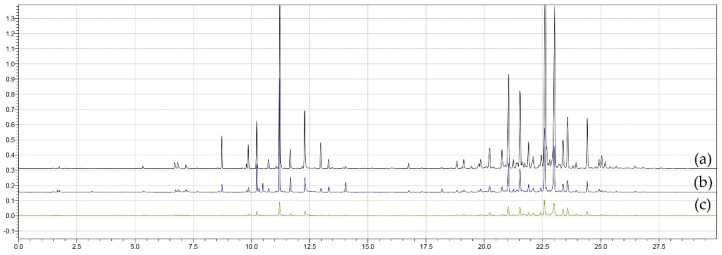
Superimposed chromatograms of treatments (**a**) T11, (**b**) T6 and (**c**) T9 obtained with the DVB/CAR/PDMS fiber in the extraction process.

**Figure 7:**
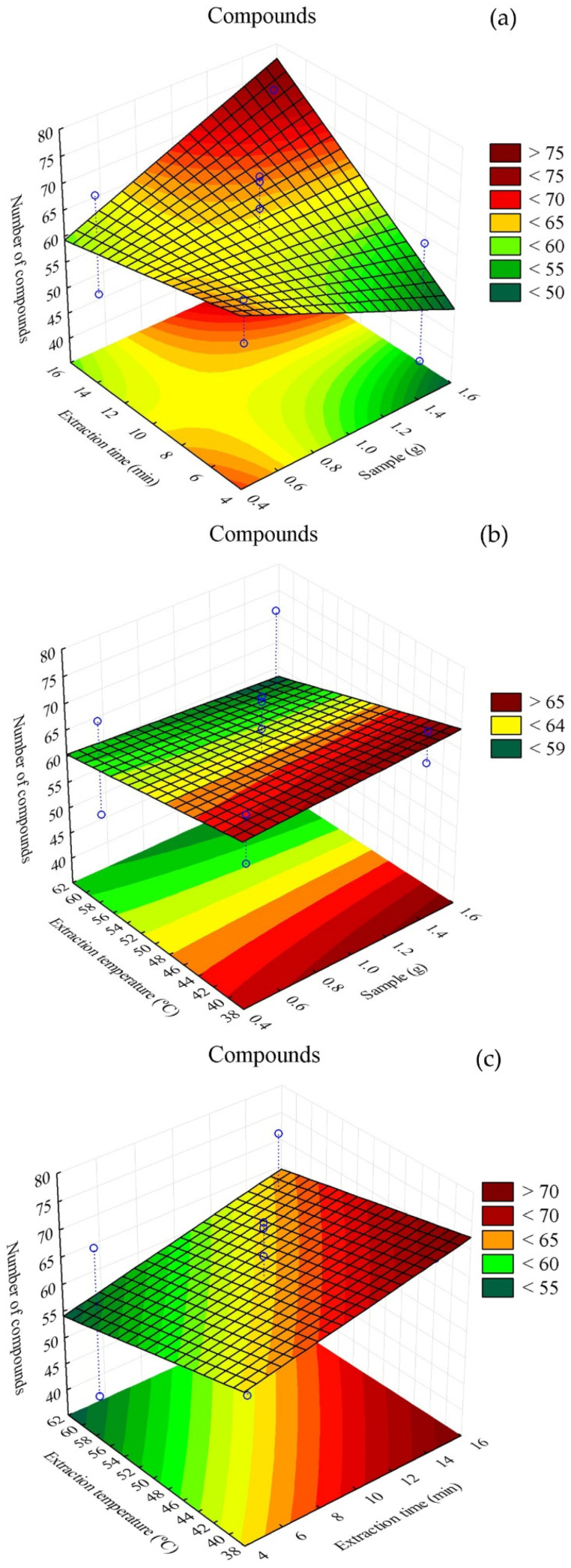
Response surface plots for volatile compounds present in alecrim-do-campo obtained with the DVB/CAR/PDMS fiber. (**a**) interaction between sample mass (g) and extraction time (min); (**b**) interaction between sample mass (g) and analyte heating temperature (°C); (**c**) interaction between fiber exposure time (min) and analyte heating temperature (°C).

Figure 6 shows overlapping chromatograms for treatments (a) T11, (b) T6 and (c) T9, obtained with the DVB/CAR/PDMS fiber and Figure 7 shows the response surface plots illustrating the interaction among factors and the number of extracted compounds. The chromatographic profiles present significant variations in intensity and number of peaks, indicating differences in volatile composition among treatments. Treatment T11 stood out for its greater diversity of extracted compounds, while T6 represented the central point of the experimental design and T9 showed the lowest number of detected compounds.

### 3.2. Identification of Compounds

Considering the criteria used for identification, 79 compounds were tentatively identified (Table 3). The fiber that showed the highest affinity for the volatiles present in the plant matrix was DVB/CAR/PDMS, with 64 adsorbed compounds, followed by CAR/PDMS and PDMS/DVB, each with 51 adsorbed compounds.

Of the compounds identified, 33 were present in all three fibers, which can be attributed to the similarity of their physical properties. Despite the overlap in VOCs, some compounds were detected in only one fiber: 10 compounds were exclusive to CAR/PDMS, 4 were exclusive to PDMS/DVB and 11 were exclusive to DVB/CAR/PDMS.

The predominant phytochemical class was that of terpenoids, accounting for 76% of the volatile profile. Among them, 14 monoterpenes and 32 sesquiterpenes were identified. This trend was observed for all fibers, but the fiber that most efficiently extracted these compounds was PDMS/DVB, which agrees with the findings of García et al. [13], concerning the affinity of this fiber for compounds belonging to these phytochemical classes.

Compounds such as α-pinene, *β*-pinene, α-thujene, sabinene, myrcene, γ-terpinene, α-copaene, α-humulene, *γ*-muurolene and *p*-cymene occur in marked amounts in other *Baccharis* species, suggesting a chemotaxonomic pattern for the genus that can be used as chemical biomarkers.

The pronounced presence of these terpenes and sesquiterpenes underscores their relevance as characteristic components of the species and highlights their potential as chemical markers and promising targets in studies on bioactivity and pharmacological applications.

## 4. Discussion

### 4.1. Effect of HS-SPME Parameters on Volatile Extraction

Evaluation of HS-SPME extraction parameters demonstrated that extraction time was the only factor exerting a statistically significant influence on volatile compound recovery when the CAR/PDMS fiber was employed. This result highlights the importance of fiber exposure time for adsorption-based coatings, particularly those containing carboxen, which require sufficient contact time to reach adsorption equilibrium for low-molecular-weight and highly volatile analytes [26]. Similar time-dependent effects have been reported in HS-SPME studies involving aromatic and medicinal plants, especially for matrices dominated by monoterpenes and light-oxygenated compounds [27].

In contrast, no significant effects of extraction time, temperature, or sample mass were observed for the PDMS/DVB and DVB/CAR/PDMS fibers. This suggests that, within the evaluated experimental ranges, these fibers provided stable extraction performance and efficient analyte partitioning between the plant matrix, headspace, and fiber coating. Mixed coatings containing divinylbenzene are known for their broader sorptive capacity and reduced sensitivity to moderate variations in extraction conditions, which may explain the absence of statistically significant factor effects [28].

Although not statistically significant, response surface analyses revealed consistent trends across fibers. Longer extraction times, particularly those exceeding 10–12 min, favored the recovery of a higher number of volatile compounds, including sesquiterpenes. This behavior can be attributed to the lower volatility and slower diffusion rates of higher-molecular-weight compounds, which benefit from extended fiber exposure [29]. Extraction temperature also influenced compound recovery in a fiber-dependent manner, with higher temperatures generally enhancing volatilization and headspace concentration [30].

### 4.2. Performance of SPME Fibers

Among the evaluated fibers, DVB/CAR/PDMS showed the highest overall compound coverage, followed by CAR/PDMS and PDMS/DVB. The superior performance of DVB/CAR/PDMS is consistent with its hybrid coating composition, which combines adsorption and absorption mechanisms and allows efficient trapping of compounds across a wide range of volatilities and polarities [27,31].

Despite similarities in total compound numbers, each fiber exhibited selectivity, as evidenced by the detection of exclusive compounds. CAR/PDMS favored lighter and more volatile constituents, including aldehydes, alcohols, and some monoterpenes, reflecting the strong affinity of carboxen for small molecules. PDMS/DVB showed affinity for several terpenoid compounds, while DVB/CAR/PDMS captured the broadest diversity of sesquiterpenes and oxygenated derivatives. These differences reinforce the importance of fiber selection based on analytical objectives, particularly when comprehensive volatilome characterization or targeted compound analysis is required [32].

The presence of 33 compounds detected by all three fibers indicates overlap in extraction capability for compounds with intermediate volatility and polarity, while the occurrence of fiber-exclusive compounds highlights the complementarity of different coatings.

### 4.3. Volatile Profile of Baccharis Dracunculifolia

A total of 79 volatile compounds were tentatively identified, with terpenoids representing approximately 76% of the volatile profile. The predominance of mono- and sesquiterpenes is consistent with previous studies on *B. dracunculifolia* essential oils and headspace analyses, which describe terpenoids as the major chemical class in this species [33]. This trend has also been reported across several species of the genus *Baccharis*, suggesting a conserved chemotaxonomic pattern [34,35].

Compounds such as α-pinene, β-pinene, limonene, γ-terpinene, β-caryophyllene, α-copaene, spathulenol, and nerolidol were consistently detected and are frequently cited in the literature as characteristic constituents of *B. dracunculifolia* [36,37,38]. The recurrent detection of these compounds across different extraction conditions and fiber coatings reinforces their relevance as chemical markers and supports their use in quality control and traceability studies.

In addition to terpenoids, the identification of aldehydes, alcohols, esters, phenylpropanoids, and aromatic nitriles contributes to the chemical complexity of the volatile profile. Although these classes represent a smaller proportion of the VOC profile, they may influence sensory attributes and ecological interactions [39].

### 4.4. Analytical and Applied Implications

Results demonstrate HS-SPME/GC-MS as a suitable and efficient technique for the analysis of volatile compounds in *B. dracunculifolia*. Compared with conventional extraction methods, HS-SPME offers advantages such as minimal sample preparation, absence of organic solvents, and reduced risk of thermal degradation, which are critical for preserving native volatile profiles [40]. The flexibility observed in extraction parameters, particularly for PDMS/DVB and DVB/CAR/PDMS fibers, further supports the applicability of this technique for routine analysis.

From an applied perspective, the dominance of terpenoids and the consistent detection of characteristic compounds across fibers highlight the potential of volatile profiling as a tool for quality assessment, authentication, and traceability of plant-derived products. The use of different fiber coatings enhances analytical coverage and reliability, supporting more robust identification of biochemical markers relevant to agroindustrial and medicinal applications.

Overall, the optimization strategy and chemical characterization presented in this study provide a solid analytical framework for future investigations focused on standardization, biomarker discovery, and the valorization of *Baccharis dracunculifolia* as a source of bioactive compounds.

## 5. Conclusions

Among the evaluated factors, only extraction time significantly influenced compound extraction using the CAR/PDMS fiber, with no specific factor or interaction effects detected for the other fibers. Extraction times exceeding 10–12 min were essential for obtaining significant numbers of extracted compounds across all fibers (CAR/PDMS, PDMS/DVB, and DVB/CAR/PDMS), regardless of temperature and sample mass, with optimal conditions varying by fiber type. Although the total number of compounds was very similar across the fibers, the CAR/PDMS and DVB/CAR/PDMS fibers showed the highest extraction efficiency. Across all experimental conditions and fiber coatings, terpenoids consistently represented the predominant class of volatile compounds detected, indicating their dominance in the volatile profile of alecrim-do-campo and their robust extractability under HS-SPME conditions.

Thus, optimizing extraction parameters is crucial for industry, providing an alternative strategy for identifying biochemical markers and ensuring the quality of plant-derived products rich in bioactive compounds. Moreover, the use of fibers composed of different materials offers substantial advantages by enabling detection across a broader spectrum of volatiles, while consistently capturing terpenoid-rich profiles, thereby enhancing the precision and reliability of biomarker searches.

## Figures and Tables

**Table 1:** Factorial design (2^3^) with three replicates at the central point.

	Levels		
Variation factors	(−)	0	(+)
Mass (g)	0.5	1	1.5
Extraction time (min)	5	10	15
Extraction temperature (°C)	40	50	60

**Table 2:** Number of compounds extracted with each fiber under different extraction conditions for volatiles present in alecrim-do-campo.

Factors				Fibers		
Treatments	Mass (g)	Time (min)	Temperature (°C)	CAR/PDMS	PDMS/DVB	DVB/CAR/PDMS
T1	0.5	5	40	54	50	59
T2	0.5	5	60	42	56	67
T3	0.5	15	40	67	51	49
T4	0.5	15	60	75	58	68
T5	1.0	10	50	60	28	73
T6	1.0	10	50	67	54	67
T7	1.0	10	50	68	33	72
T8	1.5	5	40	53	21	62
T9	1.5	5	60	41	56	39
T10	1.5	15	40	67	51	68
T11	1.5	15	60	72	67	74

**Table 3:** Tentatively identified compounds in alecrim-do-campo by HS-SPME/GC-MS.

RT (min)	Compound	MM (g mol^−1^)	MF	CAR/ PDMS	PDMS/DVB	DVB/CAR/PDMS
Alcohols						
6.8	*cis*-3-Hexen-1-ol	100	C_6_H_12_O	x	x	x
7.1	(*E*)-2-Hexen-1-ol	100	C_6_H_12_O	x	-	-
7.2	1-Hexanol	102	C_6_H_14_O	x	x	x
12.2	*α*-Phenethyl alcohol	122	C_8_H_10_O	-	-	x
13.5	*Β*-Phenethyl alcohol	122	C_8_H_10_O	-	x	x
Aldehydes						
4.4	trans-2-Pentenal	84	C_5_H_8_O	x	-	-
5.3	Hexanal	100	C_6_H_12_O	x	-	x
6.7	*trans*-Hex-2-enal	98	C_6_H_10_O	x	x	x
9.6	Benzaldehyde	106	C_7_H_6_O	x	x	x
11.7	*α*-Tolualdehyde	120	C_8_H_8_O	x	x	x
Ketone						
12.3	Acetophenone	120	C_8_H_8_O	x	x	x
Esters						
6.5	2-Butenoic acid, ethyl ester	114	C_6_H_10_O_2_	x	-	-
8.6	Hexanoic acid, methyl ester	130	C_7_H_14_O_2_	x	-	-
10.5	Hexanoic acid, ethyl ester	144	C_8_H_16_O_2_	x	-	-
14.1	Acetic acid, 2-ethylhexyl ester	172	C_10_H_20_O_2_	-	-	x
Phenylpropanoids						
17.3	3-Phenylpropanoic acid, methyl ester	164	C_10_H_12_O_2_	x	x	x
20.6	*o*-Methyl eugenol	178	C_11_H_14_O_2_	-	x	-
14.6	*cis,trans*-1,3,5-Undecatriene	150	C_11_H_18_	-	-	x
Monoterpenes						
8.6	*α*-Phellandrene	136	C_10_H_16_	x	-	-
8.7	*α*-Pinene	136	C_10_H_16_	x	-	-
9.8	*β*-Phellandrene	136	C_10_H_16_	x	x	x
9.9	*β*-Pinene	136	C_10_H_16_	x	-	-
10.2	*β*-Myrcene	136	C_10_H_16_	x	-	x
10.7	3-Carene	136	C_10_H_16_	x	x	x
10.9	4-Carene	136	C_10_H_16_	-	-	x
11.1	*o*-Cimene	134	C_10_H_14_	x	-	x
11.1	*m*-Cimene	134	C_10_H_14_	x	-	-
11.2	*D*-Limonene	136	C_10_H_16_	x	x	x
11.7	(*E*)-*β*-octimene	136	C_10_H_16_	x	-	x
12.0	*γ*-Terpinene	136	C_10_H_16_	-	-	x
12.6	2-Carene	136	C_10_H_16_	-	-	x
12.7	Terpinolene	136	C_10_H_16_	x	x	x
Oxygenated monoterpenes						
11.3	Eucalyptol	154	C_10_H_18_O	-	x	-
13.0	*β*-Linalool	154	C_10_H_18_O	x	-	x
13.9	(+)-(*E*)-Limonene oxide	152	C_10_H_16_O	-	x	x
14.9	Terpinen-4-ol	154	C_10_H_18_O	-	x	x
15.2	*α*-Terpineol	154	C_10_H_18_O	-	x	x
16.0	*cis*-Geraniol	154	C_10_H_18_O	-	x	x
Aromatic nitrile						
14.1	Benzyl nitrile	117	C_8_H_7_N	x	-	x
Sesquiterpenes						
18.6	Germacrane B	204	C_15_H_24_	-	-	x
18.6	*γ*-Elememe	204	C_15_H_24_	x	x	x
19.0	*γ*-Gurjunene	204	C_15_H_24_	x	-	-
19.1	*α*-Cubebene	204	C_15_H_24_	x	x	x
19.4	1R,4R,7R,11R-1,3,4,7-Tetramethyltricyclo [5.3.1.0(4,11)] undec-2-ene	204	C_15_H_24_	-	-	x
19.6	*α*-Amorphene	204	C_15_H_24_	x	x	-
19.8	Isoledene	204	C_15_H_24_	-	x	x
19.8	*α*-Copaene	204	C_15_H_24_	-	-	x
20.0	*α*-Cedrene	204	C_15_H_24_	-	x	x
20.1	*β*-Bourbonene	204	C_15_H_24_	x	x	x
20.2	*β*-Elememe	204	C_15_H_24_	x	x	x
20.8	*α*-Gurjunene	204	C_15_H_24_	x	x	x
20.9	*β*-Cedrene	204	C_15_H_24_	-	x	x
21.0	*β*-Caryophyllene	204	C_15_H_24_	x	x	x
21.2	*β*-Gurjunene	204	C_15_H_24_	x	x	x
21.4	*γ*-Cadinene	204	C_15_H_24_	x	x	x
21.4	(-)-*α*-Panasinsene	204	C_15_H_24_	-	-	x
21.5	(+)-Aromadendrene	204	C_15_H_24_	x	x	x
21.6	(+)-Sativene	204	C_15_H_24_	x	x	x
21.7	(E)-*β*-Farnesene	204	C_15_H_24_	-	x	x
21.9	*α*-Caryophyllene	204	C_15_H_24_	x	x	x
22.1	*γ*-Murolene	204	C_15_H_24_	x	x	x
22.3	(*E*)-Caryophyllene	204	C_15_H_24_	-	x	-
22.4	*δ*-Cadinene	204	C_15_H_24_	x	x	x
22.6	*β*-Cubebene	204	C_15_H_24_	-	x	x
22.7	*α*-Himachalene	204	C_15_H_24_	-	-	-
22.7	*β*-Eudesmene	204	C_15_H_24_	x	x	x
22.8	*β*-Patchoulene	204	C_15_H_24_	-	-	-
23.1	*β*-Himachalene	204	C_15_H_24_	-	-	x
23.8	Naphthalene, 1,2,3,4,4a,7-hexahydro-1,6-dimethyl-4-(1-methylethyl)-	204	C_15_H_24_	x	-	x
23.9	*α*-Cadinene	204	C_15_H_24_	-	x	x
25.4	*β*-Humulene	204	C_15_H_24_	-	-	-
Oxygenated sesquiterpenes						
24.3	Elemol	222	C_15_H_26_O	-	x	-
24.4	(*E*)-Nerolidol	222	C_15_H_26_O	x	-	-
24.6	Epiglobulol	222	C_15_H_26_O	-	-	x
24.8	Palustrol	222	C_15_H_26_O	x	x	x
25.0	Spathulenol	220	C_15_H_24_O	x	x	x
25.2	d-Viridiflorol	222	C_15_H_26_O	x	x	x
25.7	Ledol	222	C_15_H_26_O	-	x	x
26.8	*τ*-Cadinol	222	C_15_H_26_O	-	x	-

## Data Availability

The data supporting the findings of this study are available from the corresponding author upon request.
